# Supplementary material for: The mixed blessing of AMPK signaling in Cancer treatments
Source: BMC Cancer. 2022 Jan 25;22:105. doi: 10.1186/s12885-022-09211-1 (PMC8786626; doi:10.1186/s12885-022-09211-1)
Supplement: Supplementary file 1 — Additional file 1. [file 12885_2022_9211_MOESM1_ESM.docx]

**Table 1S.** Model equations

| $\frac{d\left[ IR \right]}{dt}=\frac{V\_pIR*\left[ pIR \right]}{Km\_pIR+[pIR]}-\frac{V\_IR*\left[ IR \right]}{Km\_IR+[IR]}$ |
| --- |
| $\frac{d\left[ pIR \right]}{dt}=\frac{V\_IR*\left[ IR \right]}{Km\_IR+[IR]}-\frac{V\_pIR*\left[ pIR \right]}{Km\_pIR+[pIR]}$ |
| $\frac{d\left[ IRS \right]}{dt}=\frac{V\_pIRS*\left[ pIRS \right]}{Km\_pIRS+[pIRS]}+\frac{V\_iIRS*\left[ iIRS \right]}{Km\_iIRS+[iIRS]}-\frac{K\_IRS\_by\_pIR*\left[ pIR \right]*[IRS]}{Km\_IRS\_by\_pIR+[IRS]}-\frac{K\_IRS\_to\_iIRS*[pmTORC1]*\left[ IRS \right]}{Km\_IRS\_to\_iIRS+[IRS]}$ |
| $\frac{d\left[ pIRS \right]}{dt}=\frac{K\_IRS\_by\_pIR*\left[ pIR \right]*[IRS]}{Km\_IRS\_by\_pIR+[IRS]}-\frac{V\_pIRS*\left[ pIRS \right]}{Km\_pIRS+[pIRS]}$ |
| $\frac{d\left[ iIRS \right]}{dt}=\frac{K\_IRS\_to\_iIRS*[pmTORC1]*\left[ IRS \right]}{Km\_IRS\_to\_iIRS+[IRS]}-\frac{V\_iIRS*\left[ iIRS \right]}{Km\_iIRS+[iIRS]}$ |
| $\frac{d\left[ AKT \right]}{dt}=\frac{V\_pAKT*\left[ pAKT \right]}{Km\_pAKT+[pAKT]}-\frac{K\_AKT\_by\_pIRS* \left[ pIRS \right]*\left[ AKT \right]}{Km\_AKT\_by\_pIRS+\left[ AKT \right]}-\frac{K\_AKT\_by\_pmTORC2* [pmTORC2]*\left[ AKT \right]}{Km\_AKT\_by\_pmTORC2+[AKT]}$ |
| $\frac{d\left[ pAKT \right]}{dt}=\frac{K\_AKT\_by\_pIRS* \left[ pIRS \right]*\left[ AKT \right]}{Km\_AKT\_by\_pIRS+\left[ AKT \right]}+\frac{K\_AKT\_by\_pmTORC2* \left[ pmTORC2 \right]*\left[ AKT \right]}{Km\_AKT\_by\_pmTORC2+\left[ AKT \right]}-\frac{V\_pAKT*\left[ pAKT \right]}{Km\_pAKT+[pAKT]}$ |
| $\frac{d\left[ mTORC1 \right]}{dt}=\frac{\left( K\_pmTORC1 +K\_pmTORC1\_by\_pAMPK*\left[ pAMPK \right] \right)*\left[ pmTORC1 \right]}{Km\_pmTORC1+[pmTORC1]}+\frac{K\_pmTORC1\_by\_pULK1*\left[ pULK1 \right]*\left[ pmTORC1 \right]}{Km\_pmTORC1\_by\_pULK1+[pmTORC1]}-\frac{K\_mTORC1\_by\_pAKT*\left[ pAKT \right]*\left[ mTORC1 \right]}{Km\_mTORC1\_by\_pAKT+[mTORC1]}-K\_mTORC1\_DEPTOR\_form*\left[ mTORC1 \right]*\left[ DEPTOR \right]+K\_mTORC1\_DEPTOR\_diss*[mTORC1\_DEPTOR]$ |
| $\frac{d\left[ pmTORC1 \right]}{dt}=\frac{K\_mTORC1\_by\_pAKT*\left[ pAKT \right]*\left[ mTORC1 \right]}{Km\_mTORC1\_by\_pAKT+[mTORC1]}-\frac{\left( K\_pmTORC1 +K\_pmTORC1\_by\_pAMPK*\left[ pAMPK \right] \right)*\left[ pmTORC1 \right]}{Km\_pmTORC1+[pmTORC1]}-\frac{K\_pmTORC1\_by\_pULK1*\left[ pULK1 \right]*\left[ pmTORC1 \right]}{Km\_pmTORC1\_by\_pULK1+[pmTORC1]}$ |
| $\frac{d\left[ mTORC2 \right]}{dt}=\frac{V\_pmTORC2*\left[ pmTORC2 \right]}{Km\_pmTORC2+\left[ pmTORC2 \right]}-\frac{K\_mTORC2\_by\_pIRS*\left[ pIRS \right]*\left[ mTORC2 \right]}{Km\_mTORC2\_by\_pIRS+\left[ mTORC2 \right]}-\frac{K\_mTORC2\_by\_pAMPK*\left[ pAMPK \right]*\left[ mTORC2 \right]}{Km\_mTORC2\_by\_pAMPK+\left[ mTORC2 \right]}-K\_mTORC2\_DEPTOR\_form*\left[ mTORC2 \right]*\left[ Deptor \right]+K\_mTORC2\_DEPTOR\_diss*[mTORC2\_Deptor]$ |
| $\frac{d\left[ pmTORC2 \right]}{dt}=\frac{K\_mTORC2\_by\_pIRS*\left[ pIRS \right]*\left[ mTORC2 \right]}{Km\_mTORC2\_by\_pIRS+\left[ mTORC2 \right]}+\frac{K\_mTORC2\_by\_pAMPK*\left[ pAMPK \right]*\left[ mTORC2 \right]}{Km\_mTORC2\_by\_pAMPK+\left[ mTORC2 \right]}-\frac{V\_pmTORC2*\left[ pmTORC2 \right]}{Km\_pmTORC2+\left[ pmTORC2 \right]}$ |
| $\frac{d\left[ mTORC1\_DEPTOR \right]}{dt}=K\_mTORC1\_DEPTOR\_form*\left[ mTORC1 \right]*\left[ DEPTOR \right]-K\_mTORC1\_DEPTOR\_diss*[mTORC1\_DEPTOR]$ |
| $\frac{d\left[ mTORC2\_DEPTOR \right]}{dt}=K\_mTORC2\_DEPTOR\_form*\left[ mTORC2 \right]*\left[ DEPTOR \right]-K\_mTORC2\_DEPTOR\_diss*[mTORC2\_DEPTOR]$ |
| $\frac{d\left[ DEPTOR \right]}{dt}=\frac{V\_pDEPTOR*\left[ pDEPTOR \right]}{Km\_pDEPTOR+\left[ pDEPTOR \right]}-\frac{K\_DEPTOR\_by\_pmTORC1*\left[ pmTORC1 \right]*[DEPTOR]}{Km\_DEPTOR\_by\_pmTORC1+[DEPTOR]}-\frac{K\_DEPTOR\_by\_pmTORC2*\left[ pmTORC2 \right]*[DEPTOR]}{Km\_DEPTOR\_by\_pmTORC2+[DEPTOR]}-K\_mTORC1\_DEPTOR\_form*\left[ mTORC1 \right]*\left[ DEPTOR \right]+K\_mTORC1\_DEPTOR\_diss*[mTORC1\_DEPTOR]-K\_mTORC2\_DEPTOR\_form*\left[ mTORC2 \right]*\left[ DEPTOR \right]+K\_mTORC2\_DEPTOR\_diss*[mTORC2\_DEPTOR]$ |
| $\frac{d\left[ pDEPTOR \right]}{dt}=\frac{K\_DEPTOR\_by\_pmTORC1*\left[ pmTORC1 \right]*[DEPTOR]}{Km\_DEPTOR\_by\_pmTORC1+[DEPTOR]}+\frac{K\_DEPTOR\_by\_pmTORC2*\left[ pmTORC2 \right]*[DEPTOR]}{Km\_DEPTOR\_by\_pmTORC2+[DEPTOR]}-\frac{V\_pDEPTOR*\left[ pDEPTOR \right]}{Km\_pDEPTOR+\left[ pDEPTOR \right]}$ |
| $\frac{d\left[ AMPK \right]}{dt}=\frac{(K\_pAMPK+K\_pAMPK\_by\_pULK1*[pULK1])*\left[ pAMPK \right]}{Km\_pAMPK+[pAMPK]}+\frac{K\_pAMPK\_by\_pmTORC1*[pmTORC1]*\left[ pAMPK \right]}{Km\_pAMPK+[pAMPK]}-\frac{(K\_AMPK+K\_AMPK\_by\_SIRT1*\left[ SIRT1 \right])*\left[ AMPK \right]}{Km\_AMPK+[AMPK]}$ |
| $\frac{d\left[ pAMPK \right]}{dt}=\frac{(K\_AMPK+K\_AMPK\_by\_SIRT1*\left[ SIRT1 \right])*\left[ AMPK \right]}{Km\_AMPK+[AMPK]}-\frac{(K\_pAMPK+K\_pAMPK\_by\_pULK1*[pULK1])*\left[ pAMPK \right]}{Km\_pAMPK+[pAMPK]}-\frac{K\_pAMPK\_by\_pmTORC1*[pmTORC1]*\left[ pAMPK \right]}{Km\_pAMPK+[pAMPK]}$ |
| $\frac{d\left[ SIRT1 \right]}{dt}=\frac{(K\_SIRT1+K\_SIRT1\_by\_pAMPK*\left[ pAMPK \right])*(\left[ SIRT1_{Total} \right]-[SIRT1])}{Km\_SIRT1+(\left[ SIRT1_{Total} \right]-[SIRT1])}-K\_SIRT1\_diss*[SIRT1]$ |
| $\frac{d\left[ ULK1 \right]}{dt}=\frac{(K\_pULK1+K\_pULK1\_by\_pmTORC1*\left[ pmTORC1 \right])*\left[ pULK1 \right]}{Km\_pULK1+[pULK1]}-\frac{\left( K\_ULK1 +K\_ULK1\_by\_pAMPK*\left[ pAMPK \right] \right)*\left[ ULK1 \right]}{Km\_ULK1+[ULK1]}$ |
| $\frac{d\left[ pULK1 \right]}{dt}=\frac{\left( K\_ULK1 +K\_ULK1\_by\_pAMPK*\left[ pAMPK \right] \right)*\left[ ULK1 \right]}{Km\_ULK1+[ULK1]}-\frac{(K\_pULK1+K\_pULK1\_by\_pmTORC1*\left[ pmTORC1 \right])*\left[ pULK1 \right]}{Km\_pULK1+[pULK1]}$ |

**Table 2S.** Model parameters**.** MM, Michaelis Menten.

| **Parameter** | **Description** | **Value** |
| --- | --- | --- |
| V_IR | Rate of activation of IR | 0.0026870 |
| Km_IR | MM constant for the activation of IR | 94.9021428 |
| V_pIR | Rate of deactivation of IR | 0.0072805 |
| Km_pIR | MM constant for the deactivation of IR | 34.9848107 |
| K_IRS_by_pIR | Rate of activation of IRS via pIR | 0.0009918 |
| Km_IRS_by_pIR | MM constant for the activation of IRS via pIR | 50.0609792 |
| V_pIRS | Rate of deactivation of IRS | 0.0051115 |
| Km_pIRS | MM constant for the deactivation of IRS | 49.9819479 |
| K_AKT_by_pIRS | Rate of activation of AKT via pIRS | 0.0003453 |
| Km_AKT_by_pIRS | MM constant for the activation of AKT via pIRS | 6.2675579 |
| K_AKT_by_pmTORC2 | Rate of activation of AKT via pmTORC2 | 0.0001568 |
| Km_AKT_by_pmTORC2 | MM constant for the activation of AKT via pmTORC2 | 5.2251572 |
| V_pAKT | Rate of deactivation of AKT | 0.0044885 |
| Km_pAKT | MM constant for the deactivation of AKT | 33.6232687 |
| K_mTORC1_by_pAKT | Rate of activation of mTORC1 via pAKT | 0.0001650 |
| Km_mTORC1_by_pAKT | MM constant for the activation of mTORC1 via pAKT | 2.3220310 |
| K_pmTORC1 | Rate of background deactivation of mTORC1 | 0.0139414 |
| K_pmTORC1_by_pAMPK | Rate of deactivation of mTORC1 via pAMPK | 0.0000066 |
| Km_pmTORC1_by_pAMPK | MM constant for the deactivation of mTORC1 | 2.1378550 |
| K_pmTORC1_by_pULK1 | Rate of deactivation of mTORC1 via pULK1 | 0.0000032 |
| Km_pmTORC1_by_pULK1 | MM constant for the deactivation of mTORC1 via ULK1 | 1.9947436 |
| K_mTORC2_by_pIRS | Rate of activation of mTORC2 via pIRS | 0.0037028 |
| Km_mTORC2_by_pIRS | MM constant for the activation of mTORC2 via pIRS | 159.9652650 |
| K_mTORC2_by_pAMPK | Rate of activation of mTORC2 via pAMPK | 0.0004358 |
| Km_mTORC2_by_pAMPK | MM constant for the activation of mTORC2 via pAMPK | 159.9223632 |
| V_pmTORC2 | Rate of deactivation of mTORC2 | 0.0070689 |
| Km_pmTORC2 | MM constant for the deactivation of mTORC2 | 8.2030495 |
| K_DEPTOR_by_pmTORC1 | Rate of activation of DEPTOR via pmTORC1 | 0.0002764 |
| Km_DEPTOR_by_pmTORC1 | MM constant for the activation of DEPTOR via pmTORC1 | 120.0534819 |
| K_DEPTOR_by_pmTORC2 | Rate of activation of DEPTOR via pmTORC2 | 0.0000775 |
| Km_DEPTOR_by_pmTORC2 | MM constant for the activation of DEPTOR via pmTORC2 | 10.5794828 |
| V_pDEPTOR | Rate of deactivation of DEPTOR | 0.0150275 |
| Km_pDEPTOR | MM constant for the deactivation of DEPTOR | 8.2347049 |
| K_mTORC1_DEPTOR_form | Rate of formation of the mTORC1-DEPTOR complex | 0.0000045 |
| K_mTORC1_DEPTOR_diss | Rate of dissociation of the mTORC1-DEPTOR complex | 0.0000334 |
| K_mTORC2_DEPTOR_form | Rate of formation of the mTORC2-DEPTOR complex | 0.0000401 |
| K_mTORC2_DEPTOR_diss | Rate of dissociation of the mTORC2-DEPTOR complex | 0.0000333 |
| K_IRS_to_iIRS | Rate of inactivation of IRS | 0.0005106 |
| Km_IRS_to_iIRS | MM constant for the inactivation of IRS | 50.1198360 |
| V_iIRS | Rate of activation of IRS from iIRS | 0.0037455 |
| Km_iIRS | MM constant for the activation of IRS from iIRS | 50.1199562 |
| K_AMPK | Rate of background activation of AMPK | 0.0278195 |
| K_AMPK_by_SIRT1 | Rate of activation of AMPK via SIRT1 | 0.0234243 |
| Km_AMPK | MM constant for the activation of AMPK | 124.2826147 |
| K_pAMPK | Rate of background deactivation of AMPK | 0.0062169 |
| K_pAMPK_by_pULK1 | Rate of deactivation of AMPK via pULK1 | 0.1314239 |
| K_pAMPK_by_pmTORC1 | Rate of deactivation of AMPK via pmTORC1 | 0.0052570 |
| Km_pAMPK | MM constant for the deactivation of AMPK | 99.9976001 |
| K_SIRT1 | Rate of background activation of SIRT1 | 0.0006433 |
| K_SIRT1_by_pAMPK | Rate of activation of SIRT1 via pAMPK | 0.0002018 |
| Km_SIRT1 | MM constant for the activation of pSIRT1 | 3.7707671 |
| K_SIRT1_diss | Rate of dissociation of SIRT1 | 0.0001394 |
| K_ULK1 | Rate of background activation of ULK1 | 0.0108759 |
| K_ULK1_by_pAMPK | Rate of activation of ULK1 via pAMPK | 0.0005689 |
| Km_ULK1 | MM constant for the activation of ULK1 | 98.9132726 |
| K_pULK1 | Rate of background deactivation of pULK1 | 0.0000365 |
| K_pULK1_by_pmTORC1 | Rate of deactivation of pULK1 via pmTORC1 | 0.0016658 |
| Km_pULK1 | MM constant for the deactivation of pULK1 | 23.5070940 |

**
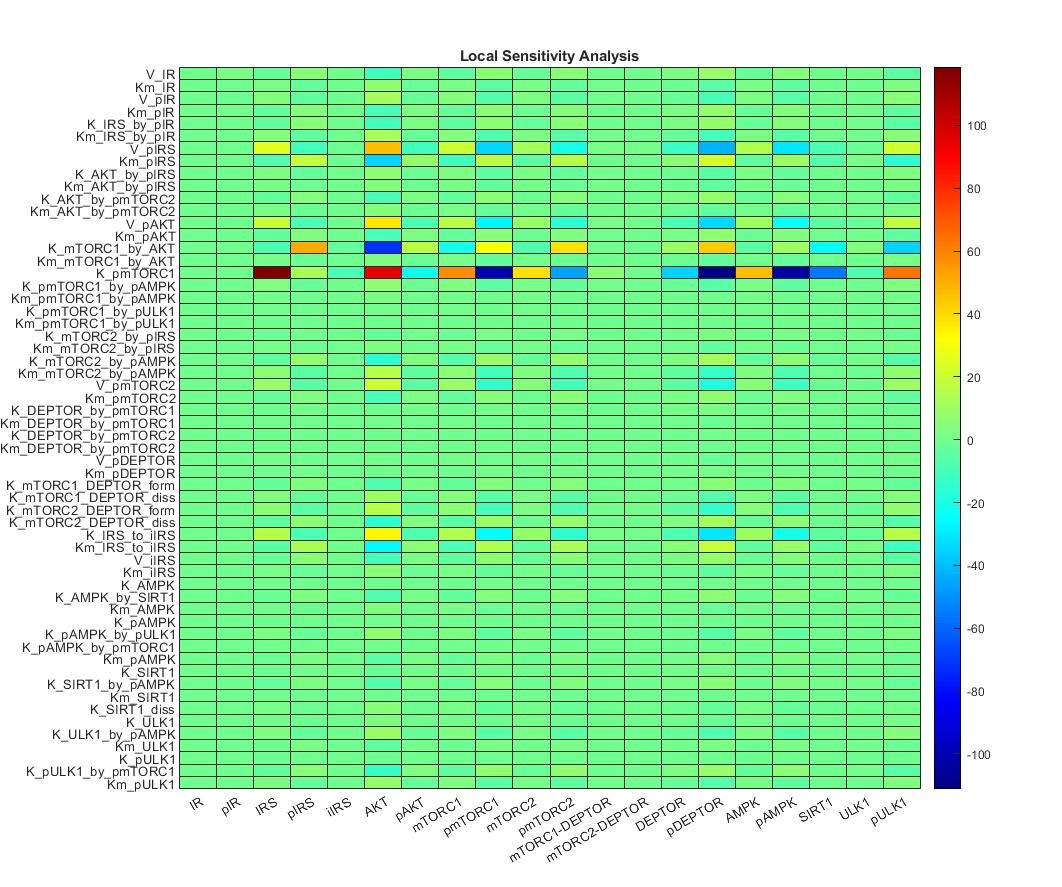
**

**Figure 1S**. Heat map that illustrates the local sensitivity of key model outputs (horizontal axis) to variations in selected model parameters (vertical axis).

**
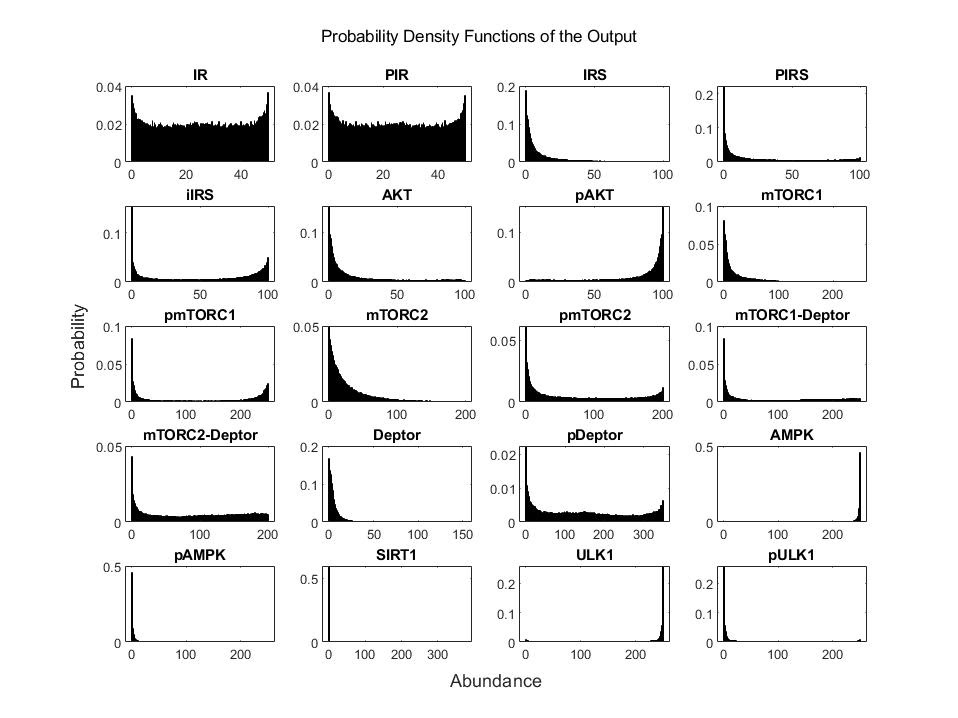
**

**Figure 2S**. Probability density functions of model outputs from the global sensitivity analysis.


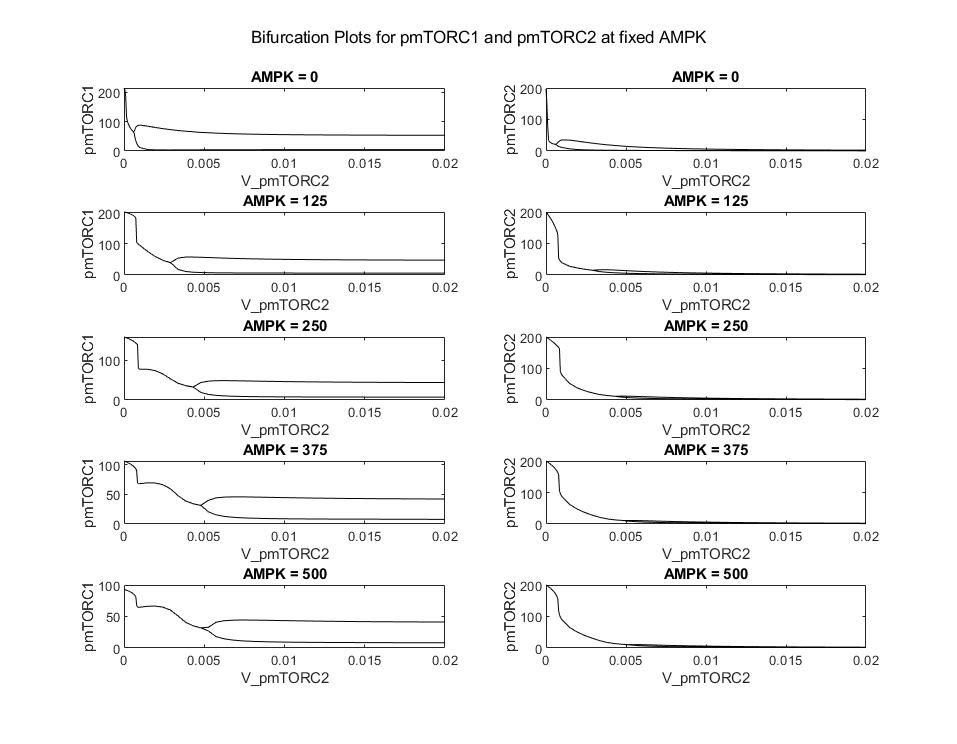


**Figure 3S**. The effect of mTORC2 inhibition and AMPK activation on cell proliferation (pmTORC1) and survival (pmTORC2).


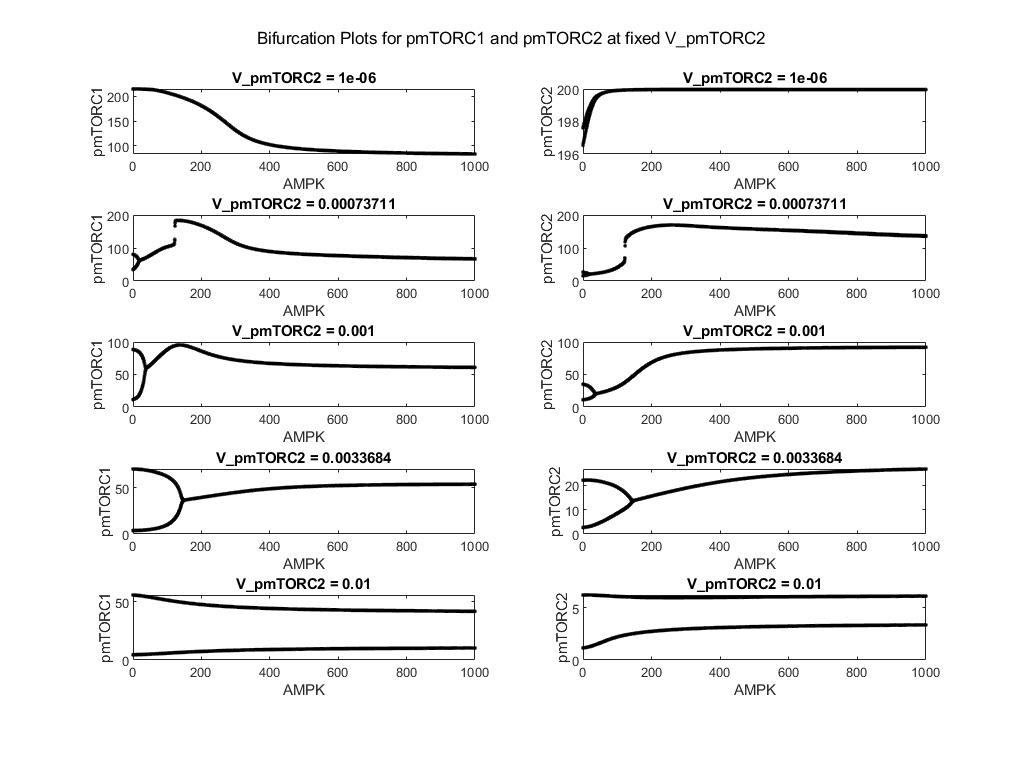


**Figure 4S**. The effect of mTORC2 inhibition and AMPK activation on cell proliferation and survival.
